# Up-regulation of VANGL1 by IGF2BPs and miR-29b-3p attenuates the detrimental effect of irradiation on lung adenocarcinoma

**DOI:** 10.1186/s13046-020-01772-y

**Published:** 2020-11-23

**Authors:** Chun-cheng Hao, Cui-yang Xu, Xin-yu Zhao, Jia-ning Luo, Gang Wang, Li-hong Zhao, Xiaofeng Ge, Xiao-feng Ge

**Affiliations:** Department of Radiotherapy, Harbin Medical University Cancer Hospital, No. 150 Haping Road, Nangang District, 150040 Harbin City, Heilongjiang China

## Abstract

Accumulating evidence suggests that radiation treatment causes an adaptive response of lung adenocarcinoma (LUAD), which in turn attenuates the lethal effect of the irradiation. Previous microarray assays manifested the change of gene expression profile after irradiation. Bioinformatics analysis of the significantly changed genes revealed that VANGL1 may notably influence the effect of radiation on LUAD. To determine the role of VANGL1, this study knocked down or overexpressed VANGL1 in LUAD. M6A level of VANGL1 mRNA was determined by M6A-IP-qPCR assay. Irradiation caused the up-regulation of VANGL1 with the increase of VANGL1 m6A level. Depletion of m6A readers, IGF2BP2/3, undermined VANGL1 mRNA stability and expression upon irradiation. miR-29b-3p expression was decreased by irradiation, however VANGL1 is a target of miR-29b-3p which was identified by Luciferase report assay. The reduction of miR-29b-3p inhibited the degradation of VANGL1 mRNA. Knockdown of VANGL1 enhanced the detrimental effect of irradiation on LUAD, as indicated by more severe DNA damage and increased percentage of apoptotic cells. Immunocoprecipitation revealed the interaction between VANGL1 with BRAF. VANGL1 increased BRAF probably through suppressing the protein degradation, which led to the increase of BRAF downstream effectors, TP53BP1 and RAD51. These effectors are involved in DNA repair after the damage. In summary, irradiation caused the up-regulation of VANGL1, which, in turn, mitigated the detrimental effect of irradiation on LUAD by protecting DNA from damage probably through activating BRAF/TP53BP1/RAD51 cascades. Increased m6A level of VANGL1 and reduced miR-29b-3p took the responsibility of VANGL1 overexpression upon irradiation.

## Background

Lung cancer is one of the most common malignant tumors. The number of newly diagnosed patients is approximately 1.8 million annually, and about 1.6 million patients die of lung cancer every year [1, 2]. Despite advancements in the diagnosis and treatment of lung cancer, patient prognosis is still poor, primarily because the pathogenesis of the disease has not been fully elucidated. Lung adenocarcinoma (LUAD) is the most common type of lung cancer and has a very poor prognosis, with a five-year survival rate below 20%. Similar to other tumors, the occurrence of LUAD is caused by multiple factors, including the environment, living habits, occupational exposure, and personal factors [3]. At present, abnormal expression of genes is considered to be the basis of the occurrence and progression of LUAD. These genes often regulate cell proliferation, survival, and radio- and chemotherapy resistance [4–6].

Radiotherapy is often used for the treatment of solid tumors. Compared with other treatment methods, radiotherapy has evident economic advantages [7–9]. Moreover, the combination of radiotherapy and other treatments usually has a better clinical outcome than individual modalities [7–9]. However, radiation treatment also causes an adaptive response of LUAD, which facilitates the development of acquired radioresistance of LUAD. Radioresistance undermines the effectiveness and efficiency of radiotherapy, resulting in treatment failure in many cases [10, 11]. Studies have found that irradiation especially at low dosages induces the up-regulation of survivin and hypoxia inducible factor in cancer cells. These adaptive responses contribute to the survival of cancers after exposing to irradiation [12, 13]. There is also evidence suggesting that radioresistance is usually associated with the overexpression of DNA repair factors (e.g., ATM) and antioxidant enzymes such as Mn-superoxide dismutase [14, 15]. Nevertheless, many scholars believe that the adaptive response to irradiation and consequently radiotherapy resistance are regulated by complex interactions between multiple genes and/or proteins. Therefore, these processes may depend on molecular and pathological processes that have not yet been identified.

6-methyladenine (m6A) is the most common RNA methylation modification in eukaryotic cells, which is regulated by m6A methyltransferases, m6A demethylases, and RNA binding proteins [16], and these are termed m6A writers, erasers, and readers, respectively. Studies in LUAD cell lines, A549 and H1299, have found that methyltransferase-like 3 (METTL3), a core m6A writer, influences the m6A levels of numerous target genes, some of which are oncogenes, such as epidermal growth factor receptor (EGFR) and Hippo pathway effector molecule TAZ [17]. Loss- and gain-of-function studies have further confirmed that METTL3 promotes the proliferation and invasion of lung cancer cells [17]. Du et al. found that microRNA-33a inhibits *METTL3* gene expression, leading to inhibition of lung cancer cell growth [18]. These results suggest that m6A modification of *METTL3* is closely related to lung cancer progression.

A class of small endogenous non-coding single-stranded RNAs, essentially consisting of miRNAs, plays important roles in mRNA expression by regulating their degradation [19–21]. Studies have found that the expression of many miRNAs in LUAD cells is aberrant after irradiation, which disrupts mRNA expression. Therefore, miRNAs, such as miR-181, miR-221, miR-222, and miR-26b, have also been associated with multiple hallmarks of cancer cells, including radiosensitivity, proliferation, migration, and invasion [19–21].

Numerous researchers have studied changes in mRNA, miRNA, and mRNA m6A levels in LUAD cells after irradiation [17, 22–24]. Based on these studies, we screened out VANGL1 and found that the upregulation of VANGL1 is associated with increased *VANGL1* mRNA m6A levels and decreased miR-29b-3p abundance in LUAD in response to irradiation. Moreover, we revealed that VANGL1 attenuated the detrimental effect of irradiation probably through regulating DNA repair signaling. This study hence added a novel understanding of the mechanism underlying the adaptive response of LUAD to irradiation.

## Materials and Methods

### Clinical study

The clinical study was approved by the Ethics Committee of Harbin Medical University, and all patients involved provided written informed consent. This study selected a total of 36 patients with unresectable stage III or IV LUAD (age range: 31–67 years) from January 2018 to May 2019. LUAD tumor staging was performed according to the International Classification of Diseases for Oncology. All these patients had not received chemotherapy, immunotherapy, and tumor surgical resection before radiotherapy. In addition, these patients had no other serious diseases before they were diagnosed with LUAD. Thoracic radiotherapy to the planning gross tumor volume (pGTV) (60-66 Gy/30‐33F/6‐7w) was delivered to patients. Patients received conventionally fractionated radiotherapy, namely 1.8-2.0 Gy irradiation each time per day, and five times each week for 6–7 weeks. Approximately 5 mL of blood was drawn before and 30 min after the final irradiation, and plasma was separated to detect miR-29b-3p levels by reverse transcriptase-quantitative polymerase chain reaction (RT-qPCR).

### Cell culture and irradiation

A549 and H1299 cells were purchased from the American Type Culture Collection (Manassas, VA, USA). A549 cells were grown in High Glucose Dulbecco’s Minimal Essential Medium (Invitrogen Life Technologies, Carlsbad, CA, USA) supplemented with 10% fetal bovine serum (FBS; HyClone, Logan, Utah, USA) and 1% penicillin/streptomycin (Invitrogen Life Technologies). H1299 cells were grown in HEPES-containing RPMI 1640 medium (HyClone) supplemented with 10% fetal bovine serum and 1% penicillin/streptomycin. Both A549 and H1299 cells were cultured at 37 °C in a humidified incubator containing a mixture of 95% air and 5% CO_2_.

A549 and H1299 cells were plated in 3.5 cm dishes and incubated in culture medium until 70–80% confluency was attained. Cells were next cultured in medium without FBS and exposed to 6 MV X-ray irradiation at a dose of 2 Gy, a dose determined based on preliminary experiments in which the cells were subjected to 1, 2, 4, and 6 Gy. Seventy-two hours after irradiation, the rates of cell proliferation and apoptosis were measured by CCK-8 assays and Alexa Fluor 488‑Annexin V /propidium iodide (PI) double-staining tests.

### Cell transfection

Tumor cells were seeded at a density of 2.0 × 10^5^ cells per well in a 6 cm dish. Lentivirus packaging VANGL1-shRNA (GenePharma, Co., Ltd. Shanghai, China) was transduced into cells to generate VANGL1-stable knockdown cell lines. Briefly, the medium was replaced with virus-containing supernatant supplemented with 10 ng/mL polybrene (Sigma-Aldrich). After 48 h, the medium was refreshed. Cells were selected by incubation with 4 mg/mL puromycin (InvivoGen). To knock-down IGF2BP2 and 3, or to over-express METTL3 and VANGL1, cells were transfected with IGF2BP2-shRNA and IGF2BP3-shRNA (GenePharma), or METTL3 and VANGL1 expression vector (pEGFP-C1; Invitrogen Life Technologies), using Lipofectamine™ 2000 (Invitrogen Life Technologies), following the procedures recommended by the manufacturers.

The inhibitors and mimics of miR-29b-3p, miR-15b-5p and miR-200a were synthesized by GenePharma, Co., Ltd. (Shanghai, China). These inhibitors were added individually to the culture media at a final concentration of 100 nM and transfected into cells using Lipofectamine™ 2000 (Invitrogen Life Technologies) according to the manufacturer’s instructions. These mimics were transfected into cells using the similar method, then these cells were exposed to 2 Gy irradiation.

### Quantitative polymerase chain reaction (qPCR)

Total cellular RNA was obtained using Trizol reagent (Invitrogen Life Technologies). Complementary DNA (cDNA) was synthesized using a First-Strand cDNA Synthesis kit (Fermentas, Vilnius, Lithuania) and 1 µg of RNA template. qPCR was performed with a 7300 Sequence Detection System (Applied Biosystems, Foster City, CA, USA) using a SYBR® Green PCR kit (Applied Biosystems). The PCR primers are shown in Table 1. U6 small nuclear RNA (U6 snRNA) and β-actin were used as an internal control to calculate the relative expression levels of miRNAs and mRNA, respectively.

**Table 1:** The primers for PCR assay

Gene name	Direction	Sequence
VANGL1	forward	5'-ATGTCACAGTCCGTGTTCCA-3'
	reverse	5'-CCAGAAGTGCCGAATCATTT-3'
METTL3	forward	5'-TTGTCTCCAACCTTCCGTAGT-3'
	reverse	5'-CCAGATCAGAGAGGTGGTGTAG-3'
IGF2BP2	forward	5'-AGCTAAGCGGGCATCAGTTTG-3'
	reverse	5'-CCGCAGCGGGAAATCAATCT-3'
IGF2BP3	forward	5'-ACGAAATATCCCGCCTCATTTAC-3'
	reverse	5'-GCAGTTTCCGAGTCAGTGTTCA-3'
beta-actin	forward	5'-CATGTACGTTGCTATCCAGGC-3'
	reverse	5'-CTCCTTAATGTCACGCACGAT-3'
miR-29b-3p	forward	5'-ACACTCCAGCTGGGTAGCACCATTTGAAATC-3'
	reverse	5'-CTCAACTGGTGTCGTGGAGTCGGCAATTCAGTTG AGAACACTGA-3'
miR-15b-5p	forward	5’-TAGCAGCACATCATGGTTTACA-3’
	reverse	5’-TGCGTGTCGTGGAGTC-3’
miR-200a-3p	forward	5ʹ-TAACACTGTCTGGTAACGATGT-3’
	reverse	5ʹ-CATCTTACCGGACAGTGCTGGA-3’
U6 snRNA	forward	5'-CTCGCTTCGGCAGCACA-3'
	reverse	5'-AACGCTTCACGAATTTGCGT-3'

### Western blot analysis

Cells and tissues were solubilized in lysis buffer (NO. 20–188; Sigma-Aldrich) and the solution was boiled for 5–10 min. The protein samples were separated by SDS-PAGE, following which the proteins were transferred onto nitrocellulose membranes. The membranes were blocked with 5% nonfat dry milk in Tris-buffered saline and then incubated with primary antibodies directed against VANGL1 (dilution 1:500, Santa Cruz Biotechnology, Inc., Santa Cruz, CA, USA), Ki67 (dilution 1:1000, Santa Cruz Biotechnology), caspase-3 (dilution 1:1000, Santa Cruz Biotechnology), BRAF (dilution 1:500, Santa Cruz Biotechnology), TP53BP1 (dilution 1:500, Santa Cruz Biotechnology), RAD51 (dilution 1:500, Santa Cruz Biotechnology), and β-actin (dilution 1:1500, Santa Cruz Biotechnology) for 2 h at room temperature. The blots were developed using peroxidase-conjugated anti-mouse IgG secondary antibody (Santa Cruz Biotechnology, Inc.), and the proteins were visualized by enhanced chemiluminescence (Amersham Biosciences, NJ, USA). β-actin was used as a loading control.

### Cell viability and apoptosis assays

Cell viability was assessed by MTT [3-(4, 5-dimethylthiazol-2-yl)-2,5-diphenyltetrazolium bromide] cell viability assays. The cells (1 × 10^3^) were seeded into 96-well plates. Optical absorbance was measured using an automated microplate reader (Tecan, NANOQUANT, Switzerland).

Cells were dual-stained with Annexin V‑Alexa Fluor 488 and propidium iodide (Kaiji Biological Inc., Nanjing, China), according to the manufacturer’s instructions. Briefly, 1 × 10^6^ cells were seeded in 6 well plate after cells were underwent transfection and radiation treatments. The cells were trypsinized and single cells suspension was mixed with 5 µL Annexin V‑Alexa Fluor 488 followed by 5 µL PI solution in dark and incubated for 15 min. The percentage of apoptotic cells was analyzed using a dual laser flow cytometer (Becton Dickinson, San Jose, CA, USA) and estimated using ModFit LT software (Verity Software House, Topsham, ME, USA).

### Cell invasion assay

Transwell chambers (Corning, New York, USA) were used to test cancer cell invasion, with 8 µm membrane filter inserts coated with Matrigel (BD Biosciences, California, USA). Briefly, the cells were trypsinized and suspended in serum-free medium. Next, 1 × 10^4^ cells were added to the upper chamber, and the lower chamber was filled with medium containing 10% FBS. After 36 h of incubation, the cells that had invaded the lower chamber were fixed with 95% ethyl alcohol for 15–20 min and stained with hematoxylin for 10 min. Next, the cells were enumerated microscopically.

### Immunofluorescence (IF) assay

Cells were fixed with 4% paraformaldehyde for 15 min and blocked with PBS containing 0.3% Triton X-100/5% BSA (w/v) for 1 h at room temperature, before incubation with antibody specific for γH2AX (1:500). Incubation with the secondary fluorescent-labeled antibody (Alexa Fluor 488, Invitrogen) was performed in the dark prior to microscopic analysis.

### M6A-IP-qPCR assay

M6A-IP-qPCR was performed following the previous study [25]. RNA was extracted and treated with DNase I to remove genomic DNA contamination. The RNA was fragmented by ultrasound for 10 s. The mRNA (~ 3 µg) as an input was then incubated with protein A magnetic beads conjugated with an anti-m6A antibody (Abcam). The magnetic beads were washed with 0.5 mL RIP Wash Buffer (Abcam), and RNA-antibody complexes were treated with protease, following which only RNA was left after the m6A antibody was digested. The precipitated mRNA and input RNA was subjected to conventional qPCR to detect the expression of VANGL1. VANGL1 m6A level = VANGL1 expression in the precipitated mRNA / its expression in the input × 100%.

### m6A dot blot assay

Total poly(A) + RNA was isolated and denatured at 65 °C within 5 min. Next, the samples were divided into subgroups of 400, 200 and 100 ng, and transferred to a nitrocellulose membrane with a Bio-Dot Apparatus (Bio-Rad, USA). The membrane was UV crosslinked for 5 min, blocked with 5% nonfat milk and incubated with specific m6A antibody (1:1000, Abcam) overnight at 4 °C. Dot-blots were incubated with HRP-conjugated anti-mouse immunoglobulin G (IgG) for 1 h before being visualized using an imaging system (Bio-Rad). The membrane was stained with 0.02% methylene blue (Sangon Biotech, Wuhan, China), which was performed to ensure consistency between the different groups.

### mRNA and protein stability assays

Actinomycin D (Sigma) was added to a concentration of 5 mg/mL to inhibit intracellular RNA synthesis. At stipulated time points, total RNA from the cells was obtained using TRIzol reagent (Invitrogen). RNA quantities were determined using RT-qPCR.

Cycloheximide (CHX, Sigma) was added to a concentration of 20 mg/mL to inhibit intracellular protein synthesis. At stipulated time points, total protein from the cells was collected for western blot assay.

## Dual-luciferase reporter assay

The putative binding site of miR-29b-3p at VANGL1 3′-UTR together with 100 nt upstream and downstream from the binding site were cloned and further inserted into into the pmirGLO vector (XhoI and NotI restriction enzyme sites; Promega, Madison, WI, USA) to construct VANGL1 wild type (WT) reporter. In addition, the putative binding site of miR-29b-3p at VANGL1 3′-UTR was mutant to construct VANGL1 mutant type (MT) reporter. A549 cells were seeded onto 12-well plates with the density of 1 × 10^4^ cells/well and transfected with the wild or mutant reporters together with either miR-29b-3p mimics or NC using Lipofectamine 2000 (Invitrogen). Cells were harvested at 48 h and the activity of firefly luciferase was normalized to that of renilla luciferase.

### Immunoprecipitation (IP) assay

Total protein was extracted and then incubated with Protein A/G Plus‑Agarose beads (Thermo Fisher Scientific) conjugated with an anti-BRAF antibody (Santa Cruz Biotechnology). After incubation at 4˚C overnight, the mixture was centrifuged at 500 × g for 5 min at 4˚C. The supernatant was discarded and the IP products were washed three times with PBS. The targeted proteins were finally detected using western blot assays.

### Tumor xenograft in nude mice

All animal experiments were approved by the Ethics Committee for Animal Research of Harbin Medical University Cancer Hospital (protocol number: 2017-029). Nude mice (4–5 weeks old, male) were purchased from the Central Animal Facility of Harbin Medical University. Two hundred milliliters of A549 cells (1 × 10^6^) were injected into the left flank of the back of each mouse. When the average tumor size reached approximately 50 mm^3^, the tumor-bearing nude mice were exposed to 2.0 Gy X-irradiation for each time. The same treatment for each group was repeated 3 times (the interval time was 5 days). Tumor volumes were measured and calculated according to the formula V = (length × width^2^)/2 (mm^3^) every 3 days. All mice were sacrificed on day 25 and the weights of tumors were measured. Primary tumors were excised, formalin-fixed, and paraffin-embedded for subsequent immunohistochemistry assays.

### Immunohistochemistry assays

Formalin-fixed and paraffin-embedded tissues of the tumor xenografts were deparaffinized and rehydrated. Tissues were treated for 20 min at 100 °C in an autoclave for antigen retrieval, and then blocked with a blocking reagent (Protein Block Serum-Free, Dako Cytomation, Glostrup, Denmark) to avoid non-specific interactions. Next, the sectioned tissues were incubated with anti-Ki67 and anti-active caspase-3 antibodies (Santa Cruz Biotechnology) overnight at 4 °C, followed by horseradish peroxidase (HRP)-labeled anti-rabbit IgG (Histofine, Simple stain MAX-PO; Nichirei, Tokyo, Japan) for 30 min at room temperature. The sections were treated with 3, 3′-diaminobenzidine tetrahydrochloride solution.

### Statistical analysis

Results are presented as the mean ± S.D. of three independent experiments. One-way analysis of variance (ANOVA) with post-hoc Dunnett’s testing was used for multiple comparisons between each group (SPSS13.0 software, IBM, USA). Paired or unpaired two-tailed t-tests were used for comparisons between two groups. Significant differences were established at *P* < 0.05.

## Results

### VANGL1 expression increased in LUAD cells following irradiation

**Fig. 1:**
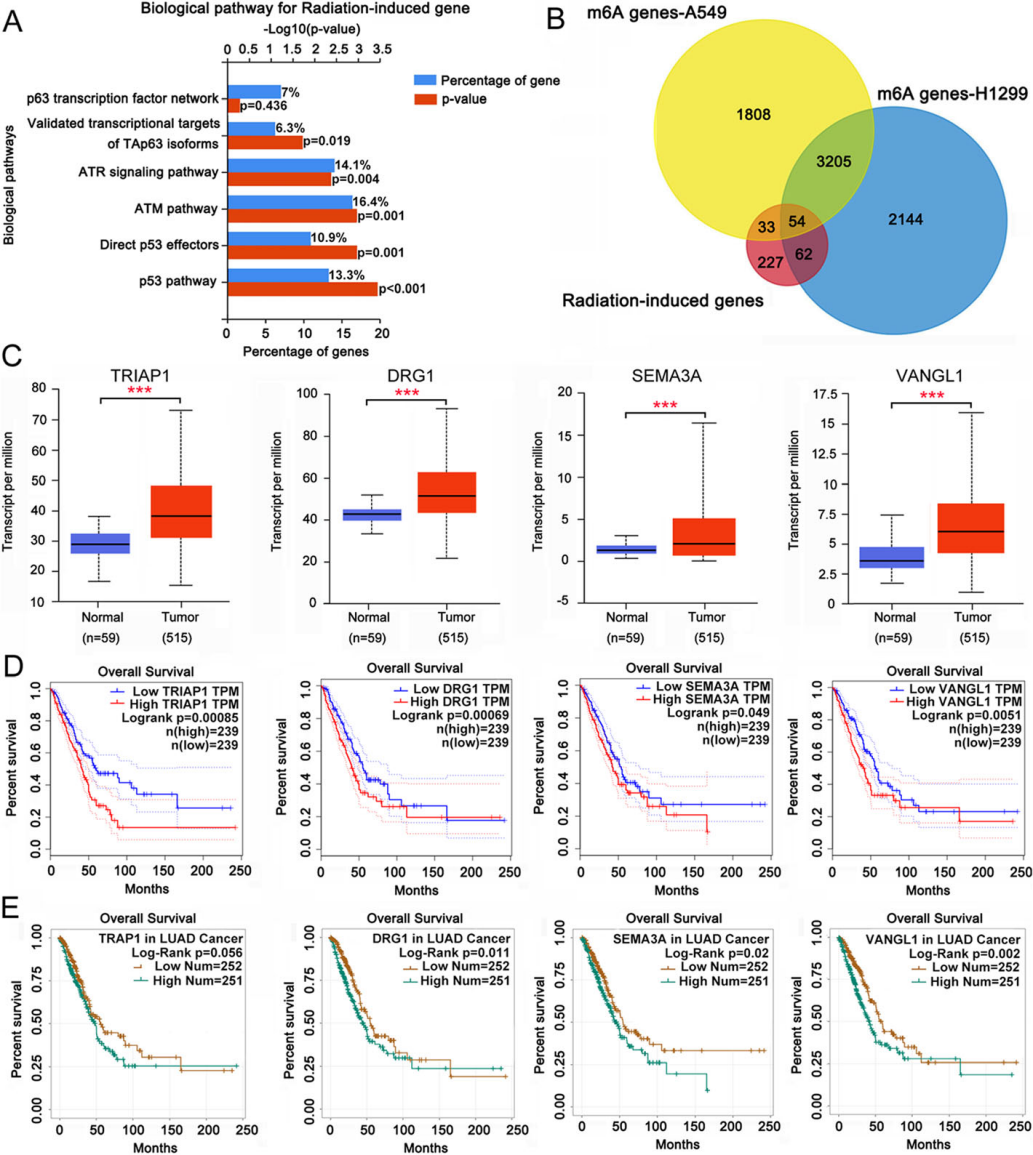
Bioinformatics analysis revealed genes that are potentially associated to radioresistance in LUAD. **a** Previous studied showed change of transcriptome profiles of radiation-resistant A549 cells under irradiation. This study performed an enrichment analysis of the significantly changed mRNA (**|**log2 fold change**|** > 1.0 and *P* < 0.05) using a functional enrichment analysis tool, FunRich 3.1.3 (http://funrich.org/index.html). **b** Previous studies also manifested that the m6A level of thousands of mRNA was affected by METTL3. This study performed an intersection analysis between the changed mRNA and METTL3-regulated mRNA. **c** TCGA dataset (http://starbase.sysu.edu.cn/index.php) showing expression levels of TRIAP1, DRG1, SEMA3A and VANGL1 in LUAD and normal lung tissues. **d** Starbase (http://starbase.sysu.edu.cn/index.php) and (E) Gepia (http://gepia.cancer-pku.cn/) showing the survival time and percentage of survival in LUAD patients with high or low TRIAP1, DRG1, SEMA3A and VANGL1 levels

Yang HJ et al. studied changes to transcriptome profiles of radiation-resistant A549 cells under irradiation [22]. We performed an enrichment analysis of significantly changed mRNAs (|log2 fold change| > 1.0 and *P* < 0.05) using a functional enrichment analysis tool, FunRich 3.1.3 (http://funrich.org/index.html). The biological pathways were ranked by the percentage of gene and then ranked by levels of –log_10_ (*p* value). The results indicated that p53 and ATM pathways were markedly related to the regulation of radiation responses in radioresistant A549 cells (Fig. 1a). These pathways are associated to DNA damage response. Previous studies also revealed that the m6A levels of thousands of mRNAs were affected by METTL3 [17]. To explore whether the changes to mRNA profiles upon irradiation were related to m6A modification, we performed intersection analysis involving the perturbed mRNAs [22] and METTL3-regulated mRNAs [17]. A total of 54 genes were observed at the intersection (Fig. 1b). Among them, four genes (*TRIAP1, DRG1, SEMA3A* and *VANGL1*) exhibited higher expression in LUAD than in normal lung tissues (Fig. 1c), according to data in TCGA dataset, as searched using Starbase (http://starbase.sysu.edu.cn/index.php). Bioinformatics analysis using Starbase and Gepia (http://gepia.cancer-pku.cn/) revealed that the high expression of these four genes was associated with poor survival rates in patients with LUAD (Fig. 1d and e).

**Fig. 2:**
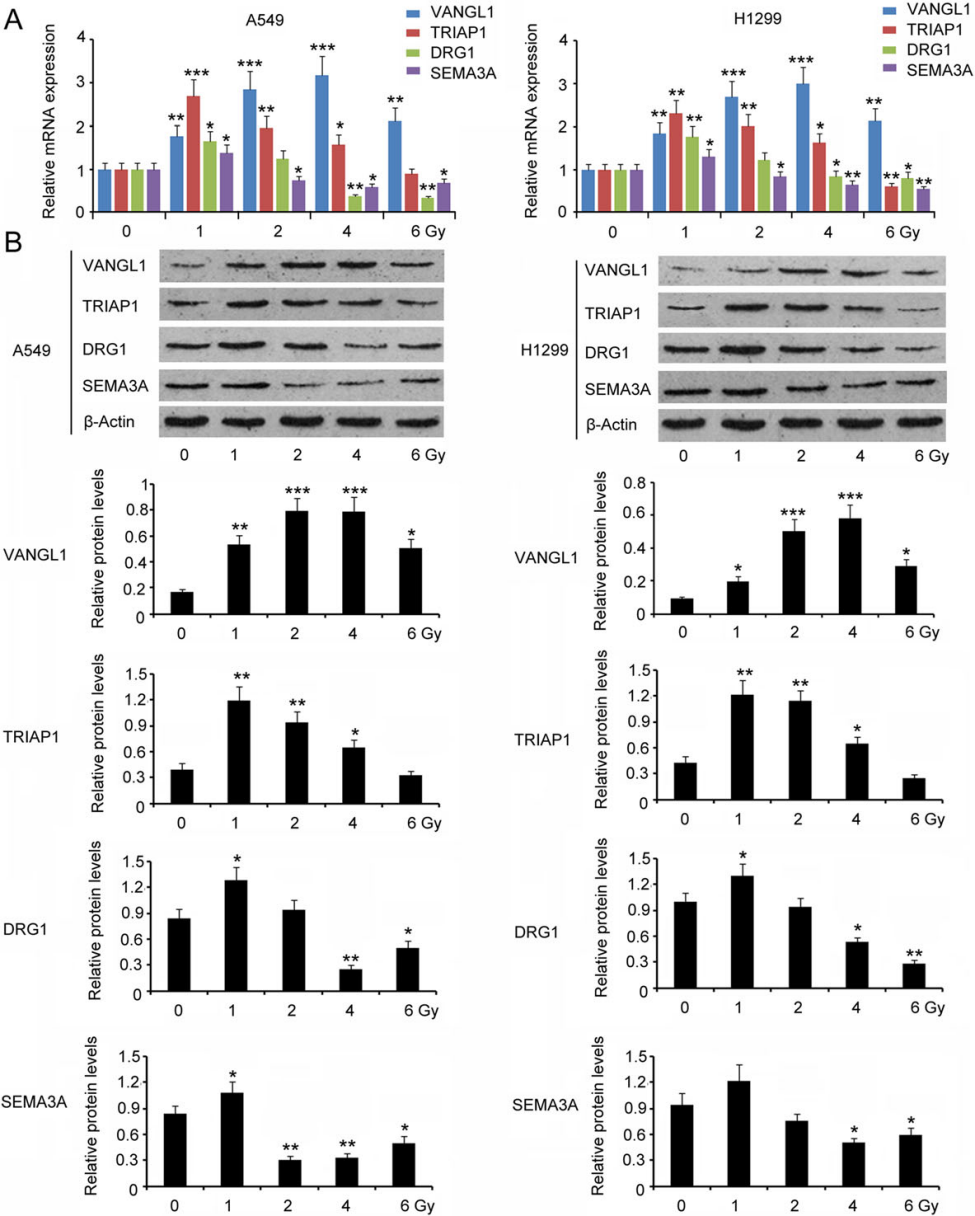
The expression of TRIAP1, DRG1, SEMA3A and VANGL1 in LUAD cells in response to irradiation. The expression of TRIAP1, DRG1, SEMA3A and VANGL1 in A549 and H1299 cells in response to different doses of irradiation was measured by PCR (**a**) and western blot assays (**b**). *P < 0.05, **P < 0.01 and ***P < 0.001 vs. 0 Gy. One-way analysis of variance (ANOVA) with post-hoc Dunnett’s testing (*n* = 3)

We further studied changes to *TRIAP1, DRG1, SEMA3A* and *VANGL1* expression in A549 and H1299 cells in response to different doses of irradiation. Both mRNA and protein levels of TRIAP1, DRG1, SEMA3A and VANGL1 were increased after exposure to 1 Gy irradiation (*P* < 0.05, *P* < 0.01 or *P* < 0.001, Fig. 2a and b). Treatment with 2 Gy irradiation further increased VANGL1 expression (*P* < 0.001), while for the other proteins, they had various degree of reduction at both mRNA and protein levels in cells after 2 Gy irradiation compared to 1 Gy irradiation. As the dose of irradiation was increased to 6 Gy, elevated expression of VANGL1 was still observed in A549 and H1299 cells (*P* < 0.01). However, TRIAP1, DRG1 and SEMA3A expression was decreased in LUAD after 6 Gy irradiation (*P* < 0.05 or *P* < 0.01). Based on these results, the role of VANGL1 in the adaptive response of LUAD to radiation treatment was further studied.

### Depletion of VANGL1 enhanced irradiation-induced injury of LUAD cells

**Fig. 3:**
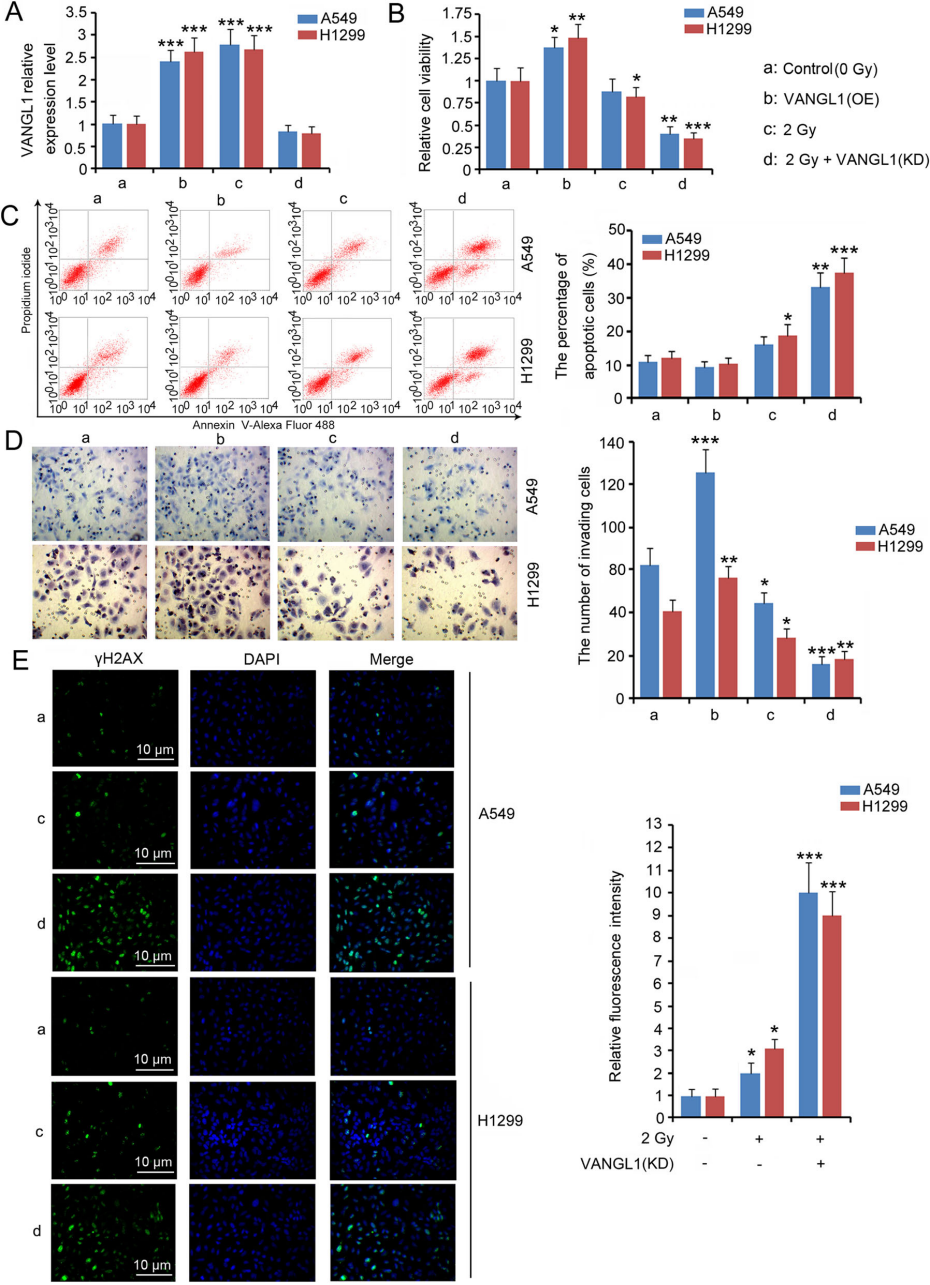
Depletion of VANGL1 sensitized LUAD cells to irradiation. VANGL1-shRNA and the overexpression vectors were transfected into LUAD cells to knock down or overexpress VANGL1. LUAD cells with VANGL1 knockdown further underwent 2 Gy irradiation. **a** PCR was performed to measured VANGL1 expression in A549 and H1299 cells. Cell viability (**b**), apoptosis rate (**c**) and invasion capacity (**d**) were also evaluated after the transfection and irradiation treatments. **e** IF was performed to determine the level of γH2AX in A549 and H1299 cells. VANGL1 overexpression alone had no effect on γH2AX level (data not shown). *P < 0.05, **P < 0.01 and ***P < 0.001 vs. control group. Unpaired two-tailed t-tests (*n* = 3)

We overexpressed VANGL1 to determine the biological role in LUAD (Fig. 3a). Moreover, VANGL1 was knocked down in LUAD prior to 2 Gy irradiation, in order to explore the role of VANGL1 in the adaptive response of LUAD to radiation treatment (Fig. 3a). Driving VANGL1 expression enhanced the viability (*P* < 0.05 or *P* < 0.01) and invasion (*P* < 0.01 or *P* < 0.001) of A549 and H1299 cells (Fig. 3b and d), yet the reduction of the percentage of apoptotic cells was moderate (Fig. 3c). This is probably because the percentage of apoptotic cells in A549 and H1299 are very low, thus VANGL1 overexpression failed to further reduced the percentage. Treatment with 2 Gy irradiation alone undermined the viability and invasion of H1299 cells and increased the percentage of apoptotic cells (*P* < 0.05, Fig. 3b, c and d), while this treatment had no effect on the viability and apoptosis of A549. VANGL1 knock-down increased the cytotoxic effects of 2 Gy irradiation on LUAD cells, because, after VANGL1 knock-down, cell viability and invasion were markedly reduced, with notably increased percentage of apoptotic cells after irradiation (*P* < 0.01 or *P* < 0.001, Fig. 3b, c and d). Irradiation-induced DNA damage is a leading cause of cell death. H2AX are specific marks of DNA double strand breaks (DSBs). When DSBs occur, the accumulation of phosphorylated H2AX (also termed γH2AX) at the DSB sites can be observed. As indicated by IF, treatment with 2 Gy irradiation increased γH2AX level (Fig. 3e). VANGL1 overexpression alone had no effect on γH2AX level (data not shown). However, VANGL1 deficiency is associated with dramatic increase of γH2AX after irradiation (*P* < 0.001, Fig. 3e).

**Fig. 4:**
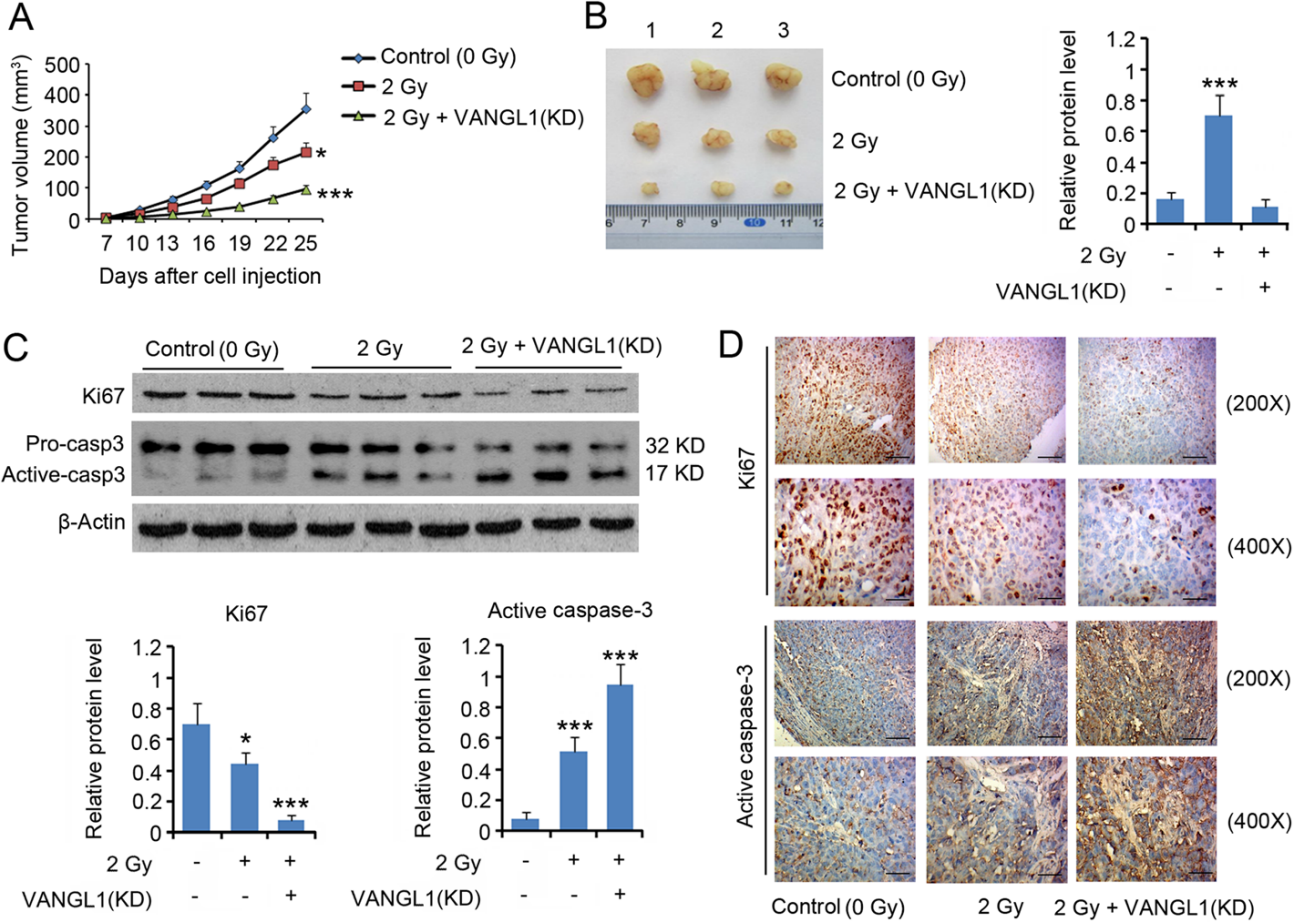
Depletion of VANGL1 suppressed the growth of LUAD tumor to irradiation. A549 cells (1 × 10^6^) with or without VANGL1 knockdown were injected into the left side on the back of each mouse. When the average tumor size reached about 50 mm^3^, the tumor-bearing nude mice were exposed to X-ray of 2.0 Gy alone for each time. The same treatment for each group was repeated 3 times (the interval time was 5 days). **a** The tumor volume was measured and calculated every 3 days. **b** All mice were killed on day 25 and the weight of tumors was measured. The primary tumors were excised for following western blot (**c**) and immunohistochemistry assays (**d**). The bars in 200 × and 400 × pictures represent 50 µm and 20 µm, respectively. *P < 0.05, **P < 0.01 and ***P < 0.001 vs. control group. Unpaired two-tailed t-tests (n = 3)

The growth of A549 in nude mice was slowed after exposure to 2 Gy X-rays (*P* < 0.05, Fig. 4a and b). VANGL1 knock-down further blocked tumor growth after exposure to the same radiation dose (*P* < 0.001). Treatment with irradiation decreased Ki67 expression, but increased active caspase-3 expression (*P* < 0.05, Fig. 4c and d). These effects of irradiation were further enhanced with VANGL1 knock-down (*P* < 0.001).

### **Elevated m6A levels associated with increased*VANGL1*mRNA stability**

**Fig. 5:**
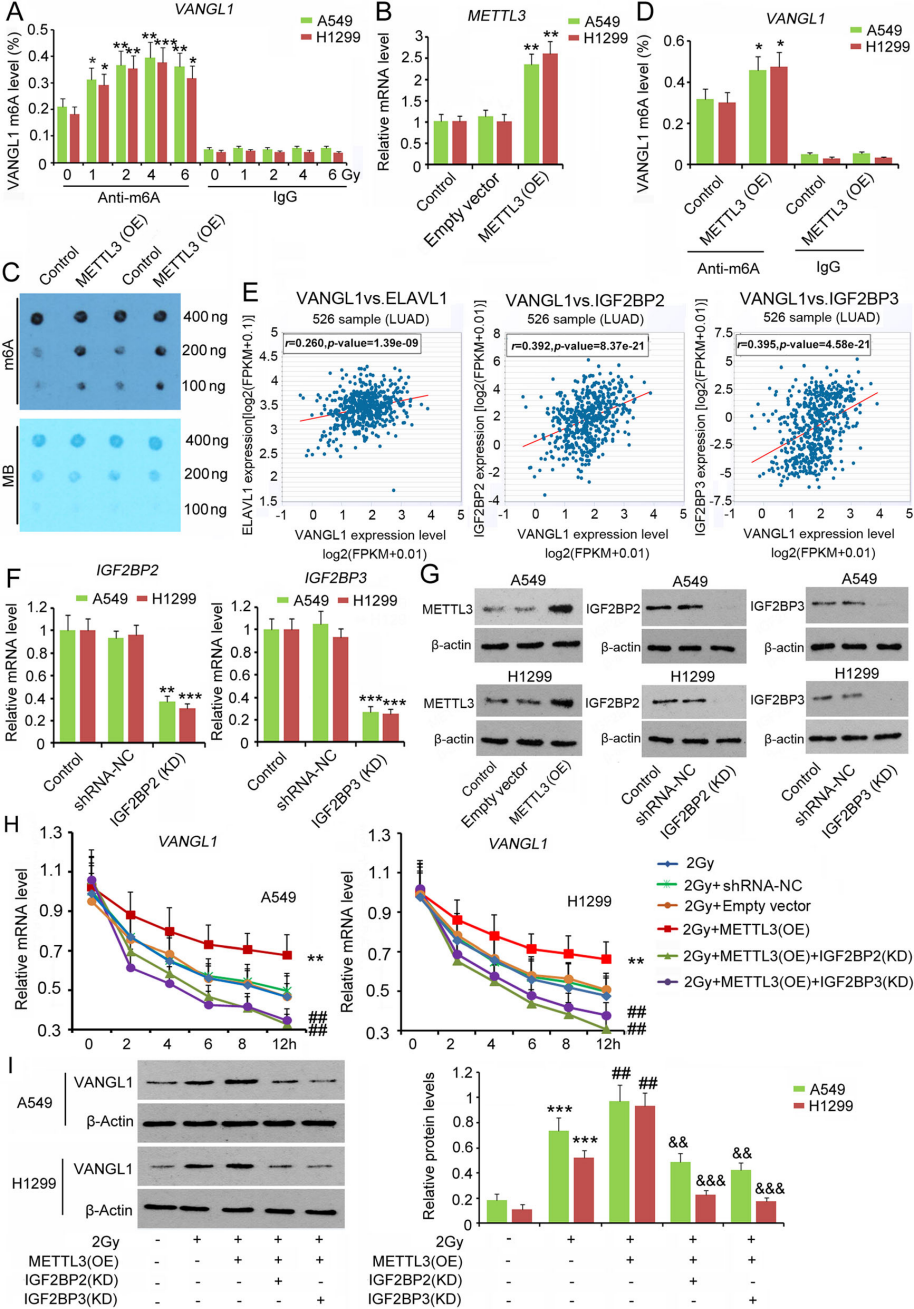
Elevated m6A level was associated to increased VANGL1 mRNA stability. **a** After irradiation from 1 to 6 Gy, m6A level of VANGL1 in A549 and H1299 cells was determined by m6A-IP-qPCR assay. *P < 0.05, **P < 0.01 and ***P < 0.001 vs. 0 Gy. One-way analysis of variance (ANOVA) with post-hoc Dunnett’s testing (*n* = 3). A549 and H1299 cells were transfected with empty vector or METTL3 expression vector, followed by (**b**) PCR detecting METTL3 mRNA level, **c** m6A dot blot detecting total m6A level and (**d**) m6A-IP-qPCR detecting m6A level of VANGL1. METTL3 (OE): the overexpression of METTL3; MB: methylene blue staining that served as a loading control. *P < 0.05 and **P < 0.01 vs. control. Unpaired two-tailed t-tests (*n* = 3). **e** Starbase web showing the correlation of VANGL1 expression with ELAVL1, IGF2BP2 and IGF2BP3 expression in LUAD. **f** PCR assay was performed to detect IGF2BP2 and IGF2BP3 expression in LUAD after transfected with IGF2BP2-shRNA and IGF2BP3-shRNA. KD: knockdown. **P < 0.01 and ***P < 0.001 vs. control. Unpaired two-tailed t-tests (n = 3). **g** Western blot assay was performed in LUAD after transfected with METTL3 expression vector, IGF2BP2-shRNA or IGF2BP3-shRNA. **h** LUAD cells were transfected with shRNA-NC, empty vector, METTL3 expression vector, IGF2BP2-shRNA and/or IGF2BP3-shRNA, followed 2 Gy irradiation. Actinomycin D was added to inhibit intracellular RNA synthesis. PCR assay was performed to determine VANGL1 mRNA level during 0 ~ 12 h after the treatments. **P < 0.01 vs. 2 Gy group. In this group, cells were only treated with 2 Gy irradiation; ^##^*P* < 0.01 vs. 2 Gy + METTL3 (OE) group. One-way analysis of variance (ANOVA) with post-hoc Dunnett’s testing (n = 3). **i** LUAD cells were transfected with METTL3 expression vector, IGF2BP2-shRNA and/or IGF2BP3-shRNA, followed 2 Gy irradiation. ***P < 0.01 vs. control group; ^##^*P* < 0.01 vs. 2 Gy group; ^&&^*P* < 0.01 and ^&&&^*P* < 0.001 vs. 2 Gy + METTL3 (OE) group. One-way analysis of variance (ANOVA) with post-hoc Dunnett’s testing (n = 3)

As indicated by m6A-IP-qPCR, m6A levels of *VANGL1* mRNA were increased after irradiation from 1 to 4 Gy in a dose-dependent manner (*P* < 0.05, *P* < 0.01 or *P* < 0.001, Fig. 5a). To understand the effects of m6A modification on VANGL1 expression, we over-expressed the m6A writer METTL3 in A549 and H1299 cells. Transfection with the expression vector increased METTL3 levels in A549 and H1299 cells (*P* < 0.01, Fig. 5b). As indicated by m6A dot blot and M6A-IP-qPCR assays, overexpression of *METTL3* also resulted in increased levels of m6A *VANGL1* mRNA (*P* < 0.05, Fig. 5c and d). Data in TCGA dataset observed via Starbase showed that *VANGL1* expression was positively correlated with the expression of *ELAVL1* (*r* = 0.26, *P* < 0.001), *IGF2BP2* (*r* = 0.392, *P* < 0.001) and *IGF2BP3* (*r* = 0.395, *P* < 0.001), three m6A readers, in particular, the latter two (Fig. 5e). Due to the high correlation between VANGL1 and IGF2BPs, this study hoped to determine the regulatory effects of IGF2BP2 and IGF2BP3 on *VANGL1* expression. Transfection with IGF2BP2-shRNA and IGF2BP3-shRNA downregulated IGF2BP2 and IGF2BP3 expression, respectively (*P* < 0.01 or *P* < 0.001, Fig. 5f and g). After exposure to 2 Gy X-irradiation, upregulation of METTL3 increased *VANGL1* mRNA stability, because the degradation of *VANGL1* mRNA over 0–12 h was reduced compared to the control group (*P* < 0.01 at 12 h, Fig. 5h). However, silencing *IGF2BP2* and *IGF2BP3* reversed the promoting effect of METTL3, resulting in rapid degradation of mRNA in comparison to the METTL3 + 2 Gy group. The level of VANGL1 protein was also increased with METTL3 overexpression (*P* < 0.05, Fig. 5i). However, the increase in VANGL1 protein abundance induced by METTL3 was reversed after *IGF2BP2* and *IGF2BP3* knockdown (*P* < 0.01 or *P* < 0.001 vs. METTL3 + 2 Gy group).

### miR-29b-3p deficiency associated with reduced ***VANGL1*** mRNA degradation

**Fig. 6:**
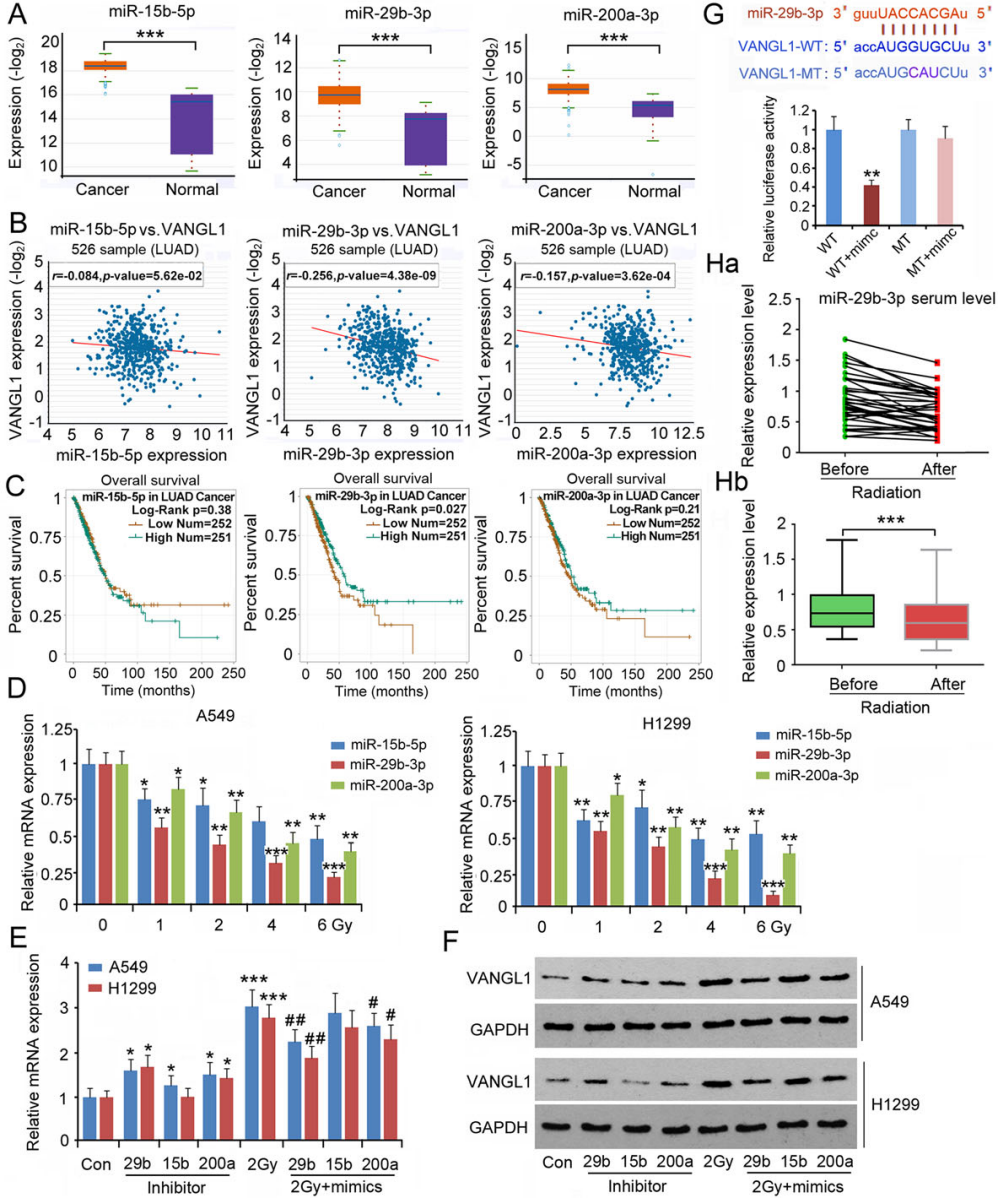
miR-29b-3p deficiency was associated with reduced degradation of VANGL1 mRNA. **a** Starbase web showing miR-15b-5p, miR-29b-3p and miR-200a-3p expression in LUAD and normal lung tissues. ***P < 0.001 vs. normal tissues. Unpaired two-tailed t-tests (*n* = 526). **b** Starbase web showing the correlation of VANGL1 expression with miR-15b-5p, miR-29b-3p and miR-200a-3p expression in LUAD. **c** Gepia web showing the survival time and percentage of survival in LUAD patients with high or low levels of miR-15b-5p, miR-29b-3p and miR-200a-3p. **d** Expression of miR-15b-5p, miR-29b-3p and miR-200a-3p was tested in LUAD cells after exposing different dosages of irradiation. *P < 0.05, **P < 0.01 and ***P < 0.001 vs. 0 Gy. One-way analysis of variance (ANOVA) with post-hoc Dunnett’s testing (*n* = 3). LUAD cells were transfected with inhibitors of miR-15b-5p, miR-29b-3p and miR-200a-3p. In addition, LUAD cells were transfected with mimics of these miRNAs, followed by 2 Gy irradiation. After these treatments, cells were subjected to (**e**) PCR and (**f**) western blot assays. *P < 0.05, and ***P < 0.001 vs. control group; ^#^*P* < 0.05, and ^##^*P* < 0.01 vs. 2 Gy group. In this group, cells were only treated with 2 Gy irradiation. One-way analysis of variance (ANOVA) with post-hoc Dunnett’s testing (*n* = 3). **g** Luciferase report assay was performed to determine whether VANGL1 is a target of miR-29b-3p. **P < 0.01 vs. WT group. Unpaired two-tailed t-tests (*n* = 3). **h** The serum level of hsa-miR-29b-3p in patients with LUAD before and after radiotherapy. Ha: the green and red dots represent the serum level of hsa-miR-29b-3p in patients with LUAD before and after radiotherapy, respectively. The green and red dots belonging to the same patient were connected by a line. Hb: the bar chart of hsa-miR-29b-3p serum level. ***P < 0.001 between before and after radiotherapy. Paired two-tailed t-tests (*n* = 36)

Using miRNA microarray assays, a study revealed changes in miRNA expression profiles after irradiation [26]. Based on this study, we identified 27 miRNAs that were predicted to target *VANGL1* from the significantly changed miRNAs (**|**log2 fold change**|** > 1.0 and *P* < 0.05) after irradiation. From these 27 miRNAs, we screened out three miRNAs, including hsa-miR-15b-5p, hsa-miR-29b-3p and hsa-miR-200a-3p, which were upregulated in LUAD tissue compared to normal lung tissue, as indicated by Starbase web (*P* < 0.001, Fig. 6a). Interestingly, these three miRNAs were down-regulated in serum after irradiation [26]. We hypothesized that these three miRNAs are upregulated in LUAD tissue, but irradiation causes their down-regulation in the tumor tissues. Expression of all three miRNAs was negatively correlated with *VANGL1* expression (Fig. 6b), but only hsa-miR-29b-3p was associated with the prognosis of LUAD (Fig. 6c). Although the expression of hsa-miR-15b-5p, hsa-miR-29b-3p and hsa-miR-200a-3p was higher in LUAD cells, their expression was dose-dependently decreased by irradiation, with the most profound reduction observed for miR-29b-3p (*P* < 0.05, *P* < 0.01 or *P* < 0.001, Fig. 6d). To further determine their regulatory effects on VANGL1 expression, we transfected LUAD cells with inhibitors and mimics of these miRNAs. Transfection with the inhibitors of miR-29b-3p and miR-200a-3p increased expression of VANGL1 in both A549 and H1299 cells (*P* < 0.05, Fig. 6e and f), whereas transfection with the miR-15b-5p inhibitor only increased VANGL1 in A549 cells (*P* < 0.05). Treatment with 2 Gy irradiation increased VANGL1 at both mRNA and protein level. However, the increase of VANGL1 induced by irradiation was suppressed with the transfection with mimics of miR-29b-3p (*P* < 0.01) and miR-200a-3p (*P* < 0.05). Based on these results, the regulatory role of miR-29b-3p in VANGL1 expression was further studied. Increasing miR-29b-3p expression decreased the relative luciferase activity of VANGL1 WT reporter, but had no effect on VANGL1 MT reporter, which suggest a direct regulatory effect of miR-29b-3p on VANGL1 (Fig. 6g). The present study collected the serum of patients with LUAD before and after the radiotherapy. After the radiotherapy, the serum levels of hsa-miR-29b-3p were decreased (*P* < 0.001, Fig. 6h), which is in agreement with the results of our *in vitro* study.

### METTL3, IGF2BP2/3 and miR-29b-3p regulate LUAD radioresistance

**Fig. 7:**
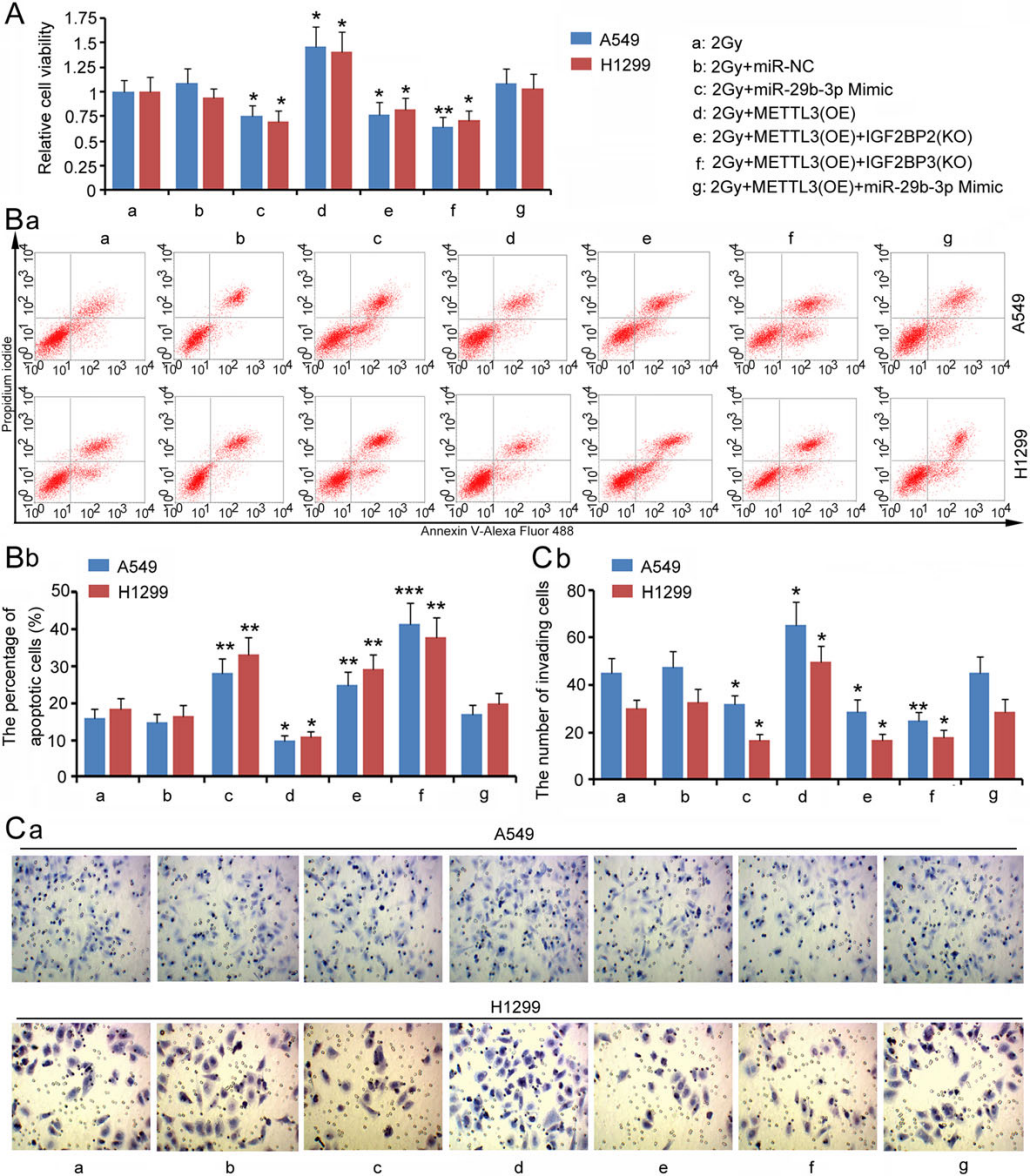
METTL3, IGF2BP2/3 and miR-29b-3p also regulated LUAD radioresistance. Before exposing to irradiation, A549 and H1299 cells were transfected with miR-29b-3p mimics, METTL3 expression vector, METTL3 expression vector + IGF2BP2 shRNA, METTL3 expression vector + IGF2BP3 shRNA, or METTL3 expression vector + miR-29b-3p mimics. After irradiation, cell viability (**a**), the percentage of apoptotic cells (**b**) and invasion capacity (**c**) were evaluated. Ba: the charts from flow cytometry measurements; Bb: the bar charts of the percentage of apoptotic cells. Ca: the charts from cell invasion assay; Cb: the bar charts of the number of invading cells.*P < 0.05, **P < 0.01 and ***P < 0.001 vs. control group. One-way analysis of variance (ANOVA) with post-hoc Dunnett’s testing (*n* = 3)

Since VANGL1 expression was regulated by METTL3, IGF2BP2/3 and miR-29b-3p, it is possible that METTL3, IGF2BP2/3 and miR-29b-3p also regulate LUAD radioresistance. Upregulation of miR-29b-3p decreased A549 and H1299 cell viability after 2 Gy irradiation (*P* < 0.05, Fig. 7a), while METTL3 overexpression elevated cell viability (*P* < 0.05). METTL3 overexpression in combination with the knock-down of IGF2BP2 or IGF2BP3 in turn suppressed A549 and H1299 cell viability after 2 Gy irradiation (*P* < 0.05). Upregulation of miR-29b-3p abolished the promoting effects of METTL3 on cell viability. Similar regulatory effects of METTL3, IGF2BP2/3 and miR-29b-3p were observed during cell invasion (Fig. 7c). Compared to cell viability and invasion, METTL3, IGF2BP2/3 and miR-29b-3p exerted opposing effects on apoptosis after irradiation (Fig. 7b).

### DNA repair signaling by BRAF/TP53BP1/RAD51 stimulated by VANGL1

**Fig. 8:**
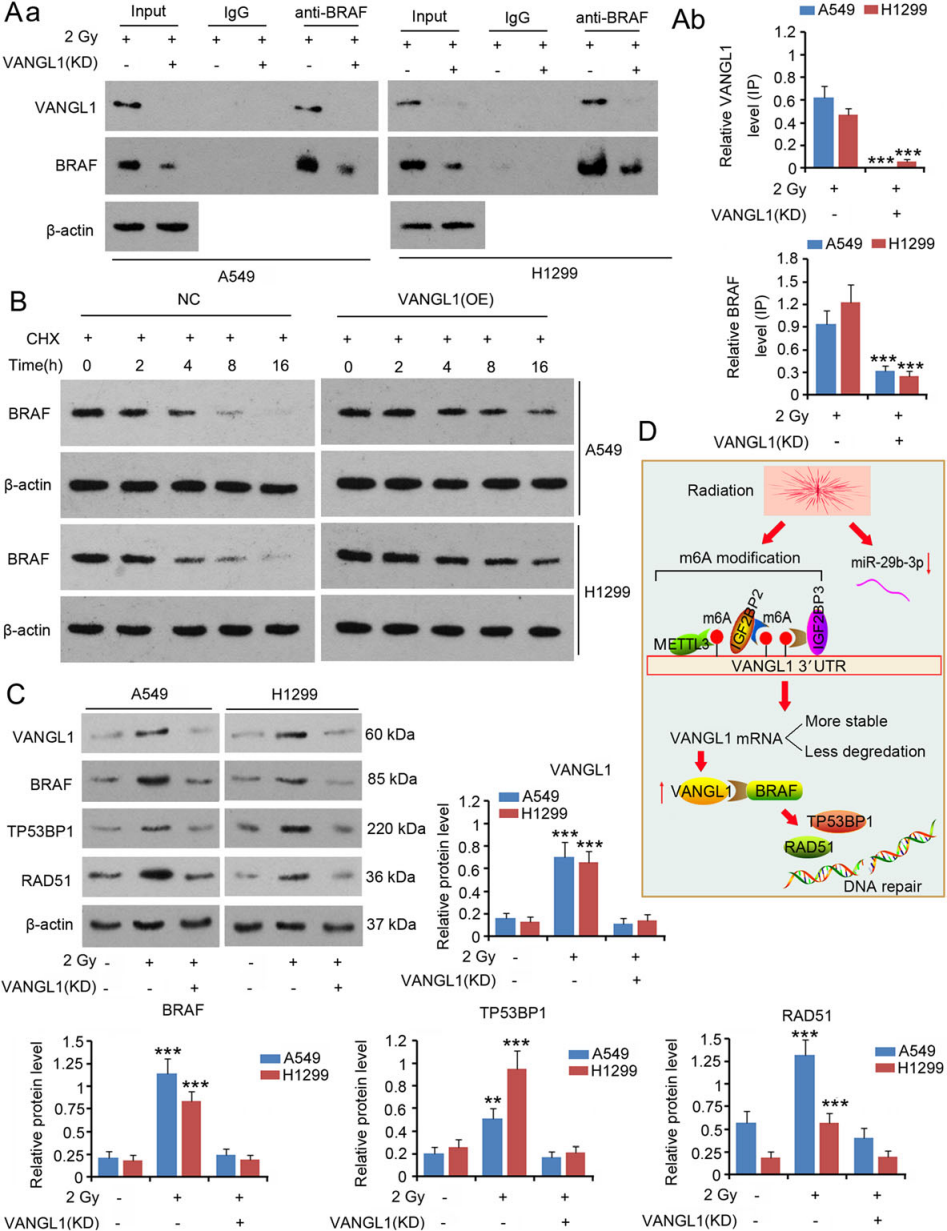
VANGL1 stimulated a DNA repair signal, BRAF/TP53BP1/RAD51. A549 and H1299 cells with or without VANGL1 knockdown were exposed to 2 Gy irradiation. **a** IP assay was performed to detect the interaction between VANGL1 and BRAF protein. Aa: western blot charts; Ab: the bar charts of protein levels. ***P < 0.001 vs. 2 Gy group. Unpaired two-tailed t-tests (*n* = 3). **b** CHX was added to block BRAF protein synthesis. Western blot assay was performed to detect BRAF protein level during 0 ~ 16 h. **c** Western blot assay was performed to detect levels of VANGL1, BRAF, TP53BP1 and RAD51 in A549 and H1299 cells. **P < 0.01 and ***P < 0.001 vs. control group. One-way analysis of variance (ANOVA) with post-hoc Dunnett’s testing (*n* = 3). **d** shows the molecular mechanism underlying VANGL1-mediated adaptive response of LUAD to irradiation. Briefly, irradiation increased the m6A level of VANGL1. IGF2BP2/3 increased VANGL1 mRNA stability in a m6A-dependent manner. Irradiation-induced deficiency of miR-29b-3p in turn increased VANGL1 expression. VANGL1 further increases the expression of BRAF and the downstream effectors, TP53BP1 and RAD51, through which VANGL1 promotes DNA repair after it is damage upon irradiation

Bioinformatics analysis (https://rnact.crg.eu/) showed a close interaction between VANGL1 and BRAF. In IP assays, VANGL1 protein was detected in BRAF protein complexes, but the enrichment of VANGL1 protein in the protein complex was decreased after VANGL1 knock-down (*P* < 0.001, Fig. 8a). We further performed a protein stability assay, in which CHX was added to block BRAF protein synthesis. Since the protein synthesis was blocked, BRAF protein level was gradually decreased by protein degradation actions (Fig. 8b). However, overexpression of VANGL1 hindered the reduction of BRAF, suggesting that VANGL1 promotes BRAF protein stability. As shown by western blot assays, 2 Gy irradiation increased the levels of VANGL1, BRAF, TP53BP1 and RAD51 (*P* < 0.001, Fig. 8c). However, the knock-down of VANGL1 also abrogated the increases in BRAF, TP53BP1 and RAD51 protein levels after 2 Gy irradiation. Figure 8d shows the molecular mechanism underlying VANGL1-mediated adaptive response of LUAD to irradiation. Briefly, irradiation increased the m6A level of *VANGL1*. IGF2BP2/3 increased *VANGL1* mRNA stability in a m6A-dependent manner. Irradiation-induced deficiency of miR-29b-3p in turn increased VANGL1 expression. The upregulation of VANGL1 protects DNA from irradiation probably through activation of the BRAF/TP53BP1 /RAD51 cascades.

## Discussion

This study found that VANGL1 expression in LUAD cells was dose-dependently increased by irradiation, while increased TRIAP1, DRG1 and SEMA3A expression after 1 Gy irradiation was not observed after higher doses of irradiation. The knock-down of VANGL1 significantly increased radiation-induced injury in LUAD cells, suggesting that VANGL1 mitigates the detrimental effect of irradiation on LUAD. VANGL1 is a transmembrane protein. It is well recognized that VANGL1 interacts with the C-terminal domain of a tumor metastasis suppressor, KAI1, therefore VANGL1 is also termed KITENIN (KAI1 C-terminal interacting tetraspanin). The cancer-promoting effects of VANGL1 on cell proliferation and invasion have been identified in colorectal and glial malignancies [27, 28]. According to our *in vitro* and *in vivo* studies, we suggested that upregulated VANGL1 protects LUAD cells from radiation-induced injury. Therefore, VANGL1 is a potential target to improve the efficiency of radiotherapy.

In this study, we observed that the m6A levels of *VANGL1* mRNA were increased by irradiation in a dose-dependent manner. Xiang et al. found that methylation at the 6 position of adenosine (m6A) in RNA is rapidly induced at sites of DNA damage in response to ultraviolet irradiation [24]. However, cells with METTL3 knock-down show delayed repair of ultraviolet-induced DNA damage and elevated sensitivity to ultraviolet, which suggests the importance of m6A modification in ultraviolet-responsive DNA damage response. To determine the effects of m6A modification on VANGL1 expression, we changed the expression of m6A writers and readers. Over-expressed METTL3 promoted *VANGL1* mRNA stability and subsequent protein levels in LUAD cells after irradiation. However, these promoting effects were reversed after IGF2BP2 and IGF2BP3 were knocked-down. These results suggested that METTL3, IGF2BP2 and IGF2BP3 collectively promoted VANGL1 expression by elevating mRNA stability.

m6A readers are a group of proteins that recognize m6A-modified RNAs and then regulate multiple processes involving RNA, such as RNA stability, translation, splicing and transport. IGF2BP2 and IGF2BP3 play important roles in mRNA stability [29, 30]. It has been identified that IGF2BPs recruit target transcripts to cytoplasmic protein-RNA complexes (mRNPs). This transcript ‘caging’ into mRNPs allows mRNA transport and transient storage, which facilitates transcripts to encounter the translational apparatus and shields them from endonuclease attacks or miRNA-mediated degradation [29, 30]. A study in colorectal carcinoma showed that higher expression of METTL3 promotes m6A levels in *SOX2* transcripts. m6A-modified *SOX2* is subsequently recognized by IGF2BP2, which prevents *SOX2* mRNA degradation [29]. Previous studies have also found that METTL3 overexpression stimulates m6A modification of hepatoma-derived growth factor (*HDGF*) mRNA. m6A reader IGF2BP3 further enhances *HDGF* mRNA stability, resulting in increased HDGF expression. HDGF contributes to the proliferation and metastasis of gastric cancer [31]. In addition, IGF2BP3 promotes *CERS6* mRNA stability, which is associated with increased expression of CERS6 and a number of malignant behaviors of breast cancer mediated by CERS6 [30]. This study found that over-expressed METTL3 mitigated the toxic effect of irradiation on LUAD cells. Knock-down of IGF2BP2 and IGF2BP3 reversed the effects of METTL3 on LUAD cells. Since METTL3, IGF2BP2 and IGF2BP3 are involved in the regulation of VANGL1 expression, their effects on radiosensitivity are probably associated with VANGL1.

The primary function of miRNAs is the induction of mRNA degradation; therefore, miRNAs are commonly associated with decreased abundance of mRNA. However, this study found that miR-29b-3p expression was decreased in LUAD cells and in serum of patients with LUAD after receiving irradiation. Using miRNA microarray technology, a number of studies have found that the expression of many miRNAs in LUAD cells and in serum of patients with LUAD are aberrantly perturbed upon irradiation [23, 26]. This suggests that radiation influences the expression of some miRNAs. Tang et al. further found that miR-208a, which is increased by X-ray irradiation, induces radioresistance via targeting p21, with corresponding activation of the AKT/mTOR pathway in lung cancer cells [26]. In contrast, miR-365 acts in a converse manner in the regulation of radiosensitivity of non-small cell lung cancer (NSCLC) cells. Upregulated miR-365 impairs the radioresistance of NSCLC cells by targeting CDC25A [32]. This study confirmed that increased miR-29b-3p enhanced the toxic effect of irradiation on LUAD. VANGL1 was shown to be the target of miR-29b-3p. miR-29b-3p deficiency resulted in the upregulation of VANGL1. However, restoration of miR-29b-3p expression blocked the upregulation of VANGL1 in LUAD cells after irradiation. These results suggested that VANGL1 mediates the effects of miR-29b-3p on radiosensitivity of LUAD cells.

The cancer-promoting effects of VANGL1 have been associated with influencing the function of KAI1, ErbB4-c-Jun and Dishevelled (Dvl)-PKC signaling. However, the mechanisms by which VANGL1 affects the adaptive response of LUAD to irradiation are unclear. It is well-recognized that DNA is vulnerable to radiation. Radiation-induced DNA damage is able to trigger cell-cycle arrest and subsequent apoptosis, which is a key mechanism responsible for radiation cytotoxicity. However, a number of studies have revealed that radiation-induced DNA damage can be repaired through DNA repair systems. Disruption to DNA repair systems notably increases radiosensitivity of cancer cells, indicating that DNA repair systems bear the responsibility for cell radioresistance. Using IP analysis, this study revealed interactions involving VANGL1 and BRAF. This interaction facilitated the increased levels of BRAF stimulated by irradiation, because overexpression VANGL1 retarded the degradation of BRAF and irradiation-induced increase in BRAF was abolished with VANGL1 knock-down. BRAF is a member of the RAF serine/threonine protein kinases family. Three RAF proteins (A, B, and C) can form homodimers and heterodimers to activate MEK/ERK signaling, promoting cell proliferation. Therefore, elimination of BRAF restrains the growth of lung cancer cells [33]. Mutant BRAF is observed in various types of cancer. BRAF mutations occur in 3–8% of patients with NSCLC, and result in increased or decreased RAF kinase activity [34]. BRAF, especially the mutant type BRAF^V600E^, has been found to enhance cancer radioresistance by promoting DNA repair by stimulating 53BP1 and RAD51 [35]. This study found that depletion of VANGL1 reduced BRAF, 53BP1 and RAD51 expression after irradiation and severe DNA damage. Therefore, the role of BRAF/53BP1/RAD51 cascades in DNA repair probably mediates the protective effects of VANGL1 against irradiation in LUAD.

There is a deficiency in this study. We did not elucidate the mechanisms through which VANGL1 facilitated increased BRAF levels after irradiation. A possible reason for this is that interactions between VANGL1 and BRAF maintain the stability of BRAF protein, which protects BRAF from ubiquitination and targeted degradation. It is also possible that the promoting effect of VANGL1 on BRAF is VANGL1-mediated signals. Additional studies are warranted to identify the mechanisms underlying the effects of VANGL1 on BRAF after irradiation.

## Conclusions

This study revealed that irradiation caused upregulation of VANGL1 levels, which, in turn, mitigated the detrimental effect of irradiation on LUAD by protecting DNA from damage probably through activating BRAF/TP53BP1/RAD51 cascades. IGF2BP2 and IGF2BP3 promoted VANGL1 stability, with increased *VANGL1* m6A levels subsequent to irradiation. Irradiation-induced deficiency of miR-29b-3p, in turn, increased VANGL1 expression. This study collectively suggested that VANGL1 is a potential candidate target in radiation therapy.

## Data Availability

The datasets generated/analyzed in the present study are available upon reasonable request from the corresponding author.

## Abbreviations

LUAD: Lung adenocarcinoma
m6A: N6 methyl adenosine
METTL3: Methyltransferase-like 3
EGFR: Epidermal growth factor receptor
pGTV: Planning gross tumor volume
RT-qPCR: Transcriptase-quantitative polymerase chain reaction
FBS: Fetal bovine serum
qPCR: Quantitative polymerase chain reactio
cDNA: Complementary DNA
IF: Immunofluorescence
IgG: Immunoglobulin G
IP: Immunoprecipitation
ANOVA: Analysis of variance
HRP: Horseradish peroxidase
DSBs: Double strand breaks.
*HDGF*: Hepatoma-derived growth factor.
Dvl: Dishevelled

## References

[1] Hirsch FR, Scagliotti GV, Mulshine JL, Kwon R, Curran WJ, Wu YL, Paz-Ares L (2017). Lung cancer: current therapies and new targeted treatments. Lancet.

[2] Lemjabbar-Alaoui H, Hassan OU, Yang YW, Buchanan P (2015). Lung cancer: Biology and treatment options. Biochim Biophys Acta.

[3] Wang J, Wang Y, Tong M, Pan H, Li D (2018). Research progress of the clinicopathologic features of lung adenosquamous carcinoma. Onco Targets Ther.

[4] Wang Y, Ding X, Liu B, Li M, Chang Y, Shen H, Xie SM, Xing L, Li Y (2020). ETV4 overexpression promotes progression of non-small cell lung cancer by upregulating PXN and MMP1 transcriptionally. Mol Carcinog.

[5] Xiao W, Wang L, Howard J, Kolhe R, Rojiani AM, Rojiani MV (2019). TIMP-1-Mediated Chemoresistance via Induction of IL-6 in NSCLC. Cancers (Basel)..

[6] Nehme E, Rahal Z, Sinjab A, Khalil A, Chami H, Nemer G, Kadara H (2019). Epigenetic Suppression of the T-box Subfamily 2 (TBX2) in Human Non-Small Cell Lung Cancer. Int J Mol Sci..

[7] Brown S, Banfill K, Aznar MC, Whitehurst P, Faivre Finn C (2019). The evolving role of radiotherapy in non-small cell lung cancer. Br J Radiol.

[8] Støchkel Frank M, Schou Nørøxe D, Nygård L, Fredberg Persson G. Fractionated palliative thoracic radiotherapy in non-small cell lung cancer - futile or worth-while? BMC Palliat Care. 2018 Jan 5;17(1):15. doi: 10.1186/s12904-017-0270-4. 10.1186/s12904-017-0270-4 PMC5756366 29304789

[9] Baker S, Dahele M, Lagerwaard FJ, Senan S (2016). A critical review of recent developments in radiotherapy for non-small cell lung cancer. Radiat Oncol.

[10] Iyengar P, Wardak Z, Gerber DE, Tumati V, Ahn C, Hughes RS, Dowell JE, Cheedella N, Nedzi L, Westover KD, Pulipparacharuvil S, Choy H, Timmerman RD (2018). Consolidative Radiotherapy for Limited Metastatic Non-Small-Cell Lung Cancer: A Phase 2 Randomized Clinical Trial. JAMA Oncol.

[11] Chang JY, Verma V, Li M, Zhang W, Komaki R, Lu C (2017). Proton Beam Radiotherapy and Concurrent Chemotherapy for Unresectable Stage III Non-Small Cell Lung Cancer: Final Results of a Phase 2 Study. JAMA Oncol.

[12] Grdina DJ, Murley JS, Miller RC, Mauceri HJ, Sutton HG, Li JJ, Woloschak GE, Weichselbaum RR (2013). A survivin-associated adaptive response in radiation therapy. Cancer Res.

[13] Gandhi NM (2018). Cellular adaptive response and regulation of HIF after low dose gamma-radiation exposure. Int J Radiat Biol.

[14] Klein C, Dokic I, Mairani A, Mein S, Brons S, Häring P, Haberer T, Jäkel O, Zimmermann A, Zenke F, Blaukat A, Debus J, Abdollahi A. Overcoming hypoxia-induced tumor radioresistance in non-small cell lung cancer by targeting DNA-dependent protein kinase in combination with carbon ion irradiation. Radiat Oncol. 2017 Dec;29(1):208. 12. 10.1186/s13014-017-0939-0 PMC5747947 29287602

[15] Matsuoka Y, Nakayama H, Yoshida R, Hirosue A, Nagata M, Tanaka T (2016). IL-6 controls resistance to radiation by suppressing oxidative stress via the Nrf2-antioxidant pathway in oral squamous cell carcinoma. Br J Cancer.

[16] Zhao W, Qi X, Liu L, Liu Z, Ma S, Wu J (2019). Epigenetic regulation of m6a modifications in human cancer. Mol Ther Nucleic Acids.

[17] Lin S, Choe J, Du P, Triboulet R, Gregory RI (2016). The m(6)A methyltransferase METTL3 promotes translation in human cancer cells. Mol Cell.

[18] Du M, Zhang Y, Mao Y, Mou J, Zhao J, Xue Q, Wang D, Huang J, Gao S, Gao Y (2017). MiR-33a suppresses proliferation of NSCLC cells via targeting METTL3 mRNA. Biochem Biophys Res Commun.

[19] Liu J, Xing Y, Rong L. miR-181 regulates cisplatin-resistant non-small cell lung cancer via downregulation of autophagy through the PTEN/PI3K/AKT pathway. Oncol Rep. 2018 Apr;39(4):1631–9. 10.3892/or.2018.6268 PMC5868400 29484437

[20] Yamashita R, Sato M, Kakumu T, Hase T, Yogo N, Maruyama E, Sekido Y, Kondo M, Hasegawa Y (2015). Growth inhibitory effects of miR-221 and miR-222 in non-small cell lung cancer cells. Cancer Med.

[21] Zang S, Zhao S, Gao X, Li Y, Zhong C, Gao J (2019). Restoration of miR-26b expression partially reverses the cisplatin resistance of NSCLC by targeting tafazzin. Onco Targets Ther.

[22] Yang HJ, Kim N, Seong KM, Youn H, Youn B (2013). Investigation of radiation-induced transcriptome profile of radioresistant non-small cell lung cancer A549 cells using RNA-sEq. PLoS One.

[23] Shin S, Cha HJ, Lee EM, Lee SJ, Seo SK, Jin HO, Park IC, Jin YW, An S (2009). Alteration of miRNA profiles by ionizing radiation in A549 human non-small cell lung cancer cells. Int J Oncol.

[24] Xiang Y, Laurent B, Hsu CH, Nachtergaele S, Lu Z, Sheng W, Xu C, Chen H, Ouyang J, Wang S, Ling D, Hsu PH, Zou L, Jambhekar A, He C, Shi Y (2017). RNA m6A methylation regulates the ultraviolet-induced DNA damage response. Nature.

[25] Huang T, Liu Z, Zheng Y, Feng T, Gao Q, Zeng W (2020). YTHDF2 promotes spermagonial adhesion through modulating MMPs decay via m6A/mRNA pathway. Cell Death Dis.

[26] Tang Y, Cui Y, Li Z, Jiao Z, Zhang Y, He Y, Chen G, Zhou Q, Wang W, Zhou X, Luo J, Zhang S (2016). Radiation-induced miR-208a increases the proliferation and radioresistance by targeting p21 in human lung cancer cells. J Exp Clin Cancer Res.

[27] Sun EG, Lee KH, Ko YS, Choi HJ, Yang JI, Lee JH, Chung IJ, Paek YW, Kim H, Bae JA, Kim KK (2017). KITENIN functions as a fine regulator of ErbB4 expression level in colorectal cancer via protection of ErbB4 from E3-ligase Nrdp1-mediated degradation. Mol Carcinog.

[28] Lee KH, Ahn EJ, Oh SJ, Kim O, Joo YE, Bae JA, Yoon S, Ryu HH, Jung S, Kim KK, Lee JH, Moon KS (2015). KITENIN promotes glioma invasiveness and progression, associated with the induction of EMT and stemness markers. Oncotarget.

[29] Li T, Hu PS, Zuo Z, Lin JF, Li X, Wu QN, Chen ZH, Zeng ZL, Wang F, Zheng J, Chen D, Li B, Kang TB, Xie D, Lin D, Ju HQ, Xu RH (2019). METTL3 facilitates tumor progression via an m6A-IGF2BP2-dependent mechanism in colorectal carcinoma. Mol Cancer.

[30] Bao G, Huang J, Pan W, Li X, Zhou T (2020). Long noncoding RNA CERS6-AS1 functions as a malignancy promoter in breast cancer by binding to IGF2BP3 to enhance the stability of CERS6 mRNA. Cancer Med.

[31] Wang Q, Chen C, Ding Q, Zhao Y, Wang Z, Chen J. METTL3-mediated m6A modification of HDGF mRNA promotes gastric cancer progression and has prognostic significance. Gut. 2019, 319639. 10.1136/gutjnl-2019-319639 31582403

[32] Li H, Jiang M, Cui M, Feng G, Dong J, Li Y, Xiao H, Fan S. MiR-365 enhances the radiosensitivity of non-small cell lung cancer cells through targeting CDC25A. Biochem Biophys Res Commun. 2019;512(2):392–8. 10.1016/j.bbrc.2019.03.082 30902389

[33] Zanucco E, El-Nikhely N, Götz R, Weidmann K, Pfeiffer V, Savai R, Seeger W, Ullrich A, Rapp UR. Elimination of B-RAF in oncogenic C-RAF-expressing alveolar epithelial type II cells reduces MAPK signal intensity and lung tumor growth. J Biol Chem. 2014;289(39):26804–16. 10.1074/jbc.M114.558999 PMC4175323 25096573

[34] Mazieres J, Cropet C, Montané L, Barlesi F, Souquet PJ, Quantin X. Vemurafenib in non-small-cell lung cancer patients with BRAFV600 and BRAFnonV600 mutations. Ann Oncol. 2020;31(2):289–94. 10.1016/j.annonc.2019.10.022 31959346

[35] Robb R, Yang L, Shen C, Wolfe AR, Webb A, Zhang X, Vedaie M, Saji M, Jhiang S, Ringel MD, Williams TM. Inhibiting BRAF oncogene-mediated radioresistance effectively radiosensitizes BRAFV600E-mutant thyroid cancer cells by constraining DNA double-strand break repair. Clin Cancer Res. 2019;25(15):4749–60. 10.1158/1078-0432.CCR-18-3625 PMC6677585 31097454

